# Biventricular versus Conduction System Pacing after Atrioventricular Node Ablation in Heart Failure Patients with Atrial Fibrillation

**DOI:** 10.3390/jcdd9070209

**Published:** 2022-07-01

**Authors:** Maja Ivanovski, Miha Mrak, Anja Zupan Mežnar, David Žižek

**Affiliations:** 1 Department of Cardiology, University Medical Centre Ljubljana, Zaloška cesta 2, 1000 Ljubljana, Slovenia; maja.ivanovski@kclj.si (M.I.); miha.mrak@kclj.si (M.M.); anja.zupanmeznar@gmail.com (A.Z.M.); 2 Faculty of Medicine, University of Ljubljana, Korytkova 2, 1000 Ljubljana, Slovenia

**Keywords:** conduction system pacing, his bundle pacing, left bundle branch pacing, biventricular pacing, AV node ablation, atrial fibrillation, heart failure

## Abstract

Conduction system pacing (CSP) modalities, including His-bundle pacing (HBP) and left bundle branch pacing (LBBP), are increasingly used as alternatives to biventricular (BiV) pacing in heart failure (HF) patients scheduled for pace and ablate strategy. The aim of the study was to compare clinical outcomes of HF patients with refractory AF who received either BiV pacing or CSP in conjunction with atrio-ventricular node ablation (AVNA). Fifty consecutive patients (male 48%, age 70 years (IQR 9), left ventricular ejection fraction (LVEF) 39% (IQR 12)) were retrospectively analysed. Thirteen patients (26%) received BiV pacing, 27 patients (54%) HBP and 10 patients (20%) LBBP. All groups had similar baseline characteristics and acute success rate. While New York Heart. Association (NYHA) class improved in both HBP (*p* < 0.001) and LBBP (*p* = 0.008), it did not improve in BiV group (*p* = 0.096). At follow-up, LVEF increased in HBP (form 39% (IQR 15) to 49% (IQR 16), *p* < 0.001) and LBBP (from 28% (IQR 13) to 40% (IQR 13), *p* = 0.041), but did not change in BiV group (*p* = 0.916). Conduction system pacing modalities showed superior symptomatic and echocardiographic improvement compared to BiV pacing after AVNA. With more stable pacing parameters, LBBP could present a more feasible pacing option compared to HBP.

## 1. Introduction

Atrio-ventricular node ablation (AVNA) with subsequent permanent pacemaker implantation provides definite rate control and represents an alternative therapeutic approach in patients with symptomatic AF and rapid ventricular rate, refractory to optimal medical treatment or catheter ablation. Meta-analysis of 21 studies showed that AVNA combined with permanent pacing may improve ventricular function, quality of life and symptoms compared with medical therapy alone [1]. However, optimal pacing modality remains unclear. Pacing from the right ventricular (RV) apex produces left ventricular (LV) dyssynchrony and potential worsening of heart failure which precludes the potential benefits of rate control after AVNA [1,2,3,4]. Furthermore, BiV pacing resulted in a significant reduction in mortality, heart failure (HF) hospitalizations, significant improvement in symptoms and significant improvement in LV remodelling. However, its benfit was much less transparent in patients with narrow QRS and LV impairment, as it still causes abnormal cardiac activation with potential worsening of electrical dyssynchrony [5,6,7,8,9].

To avoid the detrimental effects of pacing induced dyssynchrony a new concept, conduction system pacing (CSP), including His bundle Pacing (HBP) and left bundle branch pacing (LBBP), was proposed as a potential alternative. Early work from Deshmukh et al. in 2000 showed feasibility of HBP combined with AVNA [10], which was later confirmed by Vijayaraman et al. [11] and Huang et al. [12] with good short-term outcomes. In 2017, Huang et al. [13] reported the first case of LBBP technique, which overcame some of the limitations of HBP. Nonetheless, both CSP modalities offer substantial advantages over RV or BiV pacing by allowing normal infrahisian conduction, consequently providing the physiological activation, avoiding cardiac dyssynchrony and left ventricular dysfunction [14,15,16]. Although HBP is theoretically the ideal physiological pacing site, it has some inherent limitations. The implant technique is challenging and requires greater expertise in targeting a small zone. Long procedural and fluoroscopic times, high pacing capture thresholds, their rise after AVNA and consequently early battery depletions could be observed. On the other hand, LBBP showed several advantages over HBP. Since the lead is implanted in the region of the left bundle branch, which is wider extensive net-like structure and has adequate distance from the AVNA site, this modality could minimize the risk of capture threshold fluctuations after AVNA [17].

European Society of Cardiology (ESC) guidelines for the management of supraventricular tachycardia recommend AVNA with subsequent pacing (‘pace and ablate’) when tachycardia responsible for tachycardia-mediated cardiomyopathy cannot be ablated or pharmacologically controlled (Class I, level of evidence C). Recommended pacing modalities are either BIV or HBP [18]. Some observational studies already proved better clinical and echocardiographic outcomes of HBP compared to BIV in symptomatic AF patients who underwent AVNA [19]. However, no previously published study offers comparison of BIV pacing with both CSP modalities. The aim of this study was to compare clinical outcomes of BiV pacing, HBP and LBBP in HF patients with symptomatic AF and narrow QRS who underwent AVNA.

## 2. Materials and Methods

### 2.1. Study Design and Population

This single-centre, observational, retrospective study evaluated the effect of three pacing modalities on different outcomes in patients with tachycardia-induced cardiomyopathy. All consecutive patients in whom the pacemaker implantation in conjunction with AVNA was attempted between May 2015 and January 2022 were included. Patient inclusion criteria were as follows:

(a) severely symptomatic AF/atrial flutter with rapid ventricular rate, refractory to pharmacological rate or rhythm control, unsuitable for catheter ablation or in which ablation had failed;
(b) tachycardia-induced cardiomyopathy without other identifiable cause of reduced ejection fraction;
(c) ejection fraction LVEF < 50%;
(d) New York Heart Association (NYHA) class II–IV;
(e) narrow QRS complex ≤ 120 ms;
(f) the patient has provided written informed consent and was 18 years or older.

There were no pre-specified exclusion criteria. The study complied with the Declaration of Helsinki and was approved by the institutional review board. The study design was approved by the Republic of Slovenia National Medical Ethics Committee. Written informed consent was obtained from all patients.

### 2.2. Procedures

Pacemaker implantations, always preceding AVNA, were performed by three experienced operators. Defibrillator backup was implanted at the discretion of the physician according to the ESC guidelines [20].

#### 2.2.1. Biventricular Pacing

Implantation of the BiV device was performed using standard techniques. The RV lead was positioned in the RV apex or septum. As BiV pacing effectiveness relies on the positioning of the left ventricular lead, we always performed venography and targeted the most appropriate CS tributary, preferably posterolateral or lateral vein. The atrial port of the device was isolated. Any commercially available biventricular pacemaker and leads were permitted. After the procedure, V-V delay was optimised to achieve the narrowest possible QRS complex [8].

#### 2.2.2. His Bundle Pacing

The procedure was performed as previously described [11,19,21]. In short, a 4.1 Fr bipolar active fixation lead (SelectSecure 3830, Medtronic, Minneapolis, MN, USA) and dedicated delivery sheath (C315His or C304, Medtronic, Minneapolis, MN, USA) were used for His bundle area mapping under fluoroscopy. To locate the anatomical landmark of His bundle area, visualisation of the tricuspid valve annulus via contrast injection through delivery sheath was usually performed before mapping [22]. The lead was then advanced through the sheath for unipolar mapping using the electrophysiological system LAB system Pro, BARD (Boston Scientific, Lowell, MA, USA) or EP-TRACER 2 Portable (CardioTek B.V., Sittard, The Netherlands) at a sweep speed of 100 mm/s. After localising the His bundle potential, preferably the most distal His deflection with ventricular to atrial electrogram ratio >3:1, pacing was attempted before the lead fixation to confirm HB capture. Acute HBP threshold ≤2.5 V at 1 ms was considered acceptable. HBP lead was connected to the atrial port of the dual-chamber device, and additional backup RV lead or implantable cardioverter-defibrillator (ICD) lead was connected to the ventricular port of the device, usually programmed in DDD mode. In one patient, we implanted an atrial lead to allow the option of establishing sinus rhythm and atrioventricular coupling during follow-up.

#### 2.2.3. Left Bundle Branch Pacing

The procedural steps for delivering LBBP were previously reported [17,23]. LBBP was attempted with two different combinations of pacing leads and delivery sheaths. The Medtronic 4.1 Fr bipolar active fixation lead (SelectSecure 3830, Medtronic, Minneapolis, MN, USA) was delivered through a long preshaped sheath (C315His or C304, Medtronic, Minneapolis, MN, USA). Some implantations were performed with a 5.6 Fr stylet-driven pacing lead (Solia S60, Biotronik, Berlin, Germany) delivered through a preshaped sheath (Selectra 3D-55-39, Biotronik, Berlin, Germany). Additionally, electrophysiological system LAB system Pro, BARD (Boston Scientific, Lowell, MA, USA) or EP-TRACER 2 Portable (CardioTek B.V., Sittard, The Netherlands) at a sweep speed of 100 mm/s was used. The fluoroscopic HBP location was located as described above and set as a marker for LBBP lead implantation. The LBBP lead was positioned approximately 1–1.5 cm distal to the HBP lead position in the RV septum along the line between the HBP site and RV apex. We sought a paced QRS morphology with a “w” pattern in lead V1 before screwing the lead deep into the interventricular septum. The suitable lead position was confirmed by fluoroscopic fulcrum sign, paced morphology of right bundle branch block (RBBB) pattern and occurrence of premature ventricular beats with RBBB morphology. Given that the pacing parameters with LBBP are typically low and stable, backup RV lead was not implanted. 

#### 2.2.4. Atrioventricular Node Ablation

**Figure 1:**
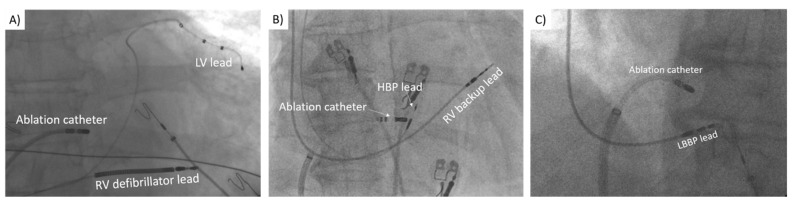
Fluoroscopic view of the ablation catheter in relation to biventricular pacing (**A**), His bundle pacing (**B**) and left bundle branch pacing (**C**) lead.

AVNA was performed following pacemaker implantation. The technique has been described previously [12,15,17,19]. The lower pacing rate was temporarily set to 40 bpm for the duration of the procedure. A long sheath (SR0; Fast-CathTM, Abbott, Abbott Park, IL, USA) was inserted through the femoral vein to the atrioventricular junction region. A 4- or 3.5-mm irrigated tip ablation catheter (FlexabilityTM, Abbott, Abbott Park, IL, USA or CelsiusVR ThermocoolVR, Biosense Webster, Irvine, CA, USA) was used to perform AVNA. Right anterior oblique or anteroposterior view was used for positioning of the catheter tip to the presumed area of the AV node in the mid-septum. The location was optimised according to the intracardiac electrograms. In the case of HBP, the ring electrode of the HBP was usually targeted. This provided a safe distance that prevented the threshold rise during or following AVNA due to the vicinity of the HBP lead tip. Immediately after complete heart block was achieved, pacing started from HBP lead at 0.5 V above the capture threshold to enable rapid detection of any rise in His capture threshold. An abrupt drop of heart rate to 40 bpm signified successful AVNA, which was continued for 60 s thereafter. Any peri-procedural lead dislocation, rise or loss of pacing capture threshold were documented. Fluoroscopic view of the ablation catheter in relation to the pacemaker lead position can be seen in Figure 1.

Following AVNA, the lower pacing rate was initially set to 90 bpm and decreased to 70 bpm at 1-month follow-up.

### 2.3. Outcomes and Follow-Up

Implant success rate, implant characteristics and procedural complications were documented. Device interrogation was performed at each follow-up visit (1 month, 6 months and annually), latest pacing parameters were used for the analysis. Lead-related complications, including infection, dislodgement, rise of pacing threshold, loss of capture and early battery depletion, were also tracked during follow-up. Clinical assessment data, including echocardiography, clinical and laboratory evaluation, was performed at baseline and approximately 6 months thereafter.

### 2.4. Statistical Analyses

Categorical variables were expressed as relative counts and percentages. Fisher exact test was used to examine the differences between the groups. Continuous variables were reported as mean ± standard deviation or median (interquartile range), according to the distribution. For continuous variables independent 2-sample t-tests were performed to compare the differences between the two groups, and paired t-tests were used to compare the differences between baseline and follow-up data if they were normally distributed. Otherwise, Mann-Whitney U tests for between-group comparisons or Wilcoxon signed-rank tests for within-group comparisons were used to assess the above-mentioned differences. To compare the differences between all three groups, ANOVA or Kruskal-Wallis test were utilised, as appropriate. All hypotheses were two-tailed, and *p*-value ≤ 0.05 was considered significant. Data management and statistical analyses were performed using IBM SPSS Statistics 22.0 SPSS Statistics (Version 25.0., Armonk, NY, USA).

## 3. Results

### 3.1. Patient Characteristics

Fifty consecutive patients in whom CSP or BIV pacing combined with AVNA was attempted were included in the study. Twelve patients received BIV pacing, 28 HBP and 10 were LBBP recipients. Baseline characteristics are presented in Table 1 and did not differ between the three groups. Median age of the study population was 70 years (66–75), 24 (48%) of the patients were male. Median NYHA class was 3, mean baseline QRS width 100 ms (±13), median LVEF 39% (30–42) and averaged left atrial volume index (LAVI) 58 mL/m2 (±14). Permanent atrial flutter was present in 22% of the patients. Before the procedure HF therapy was optimized and did not change during follow-up. Median follow-up time was 5 months (3.5–6) in BIV group, 6 months (5–13) in HBP group and 2 months (1–3.25) in LBBP group.

### 3.2. Procedural Outcomes

All device implantations and subsequent AVNAs were acutely successful. Most of the procedures were de-novo implantations. Only one upgrade procedure with HBP was performed in HF patient with previous ICD implantation. ICD was implanted in 3 BIV, 2 HBP patients and 3 patients in LBBP group (*p* = 0.170). All ICDs were implanted for primary prevention. Pacemaker implantation fluoroscopy time was significantly shorter in both CSP groups (BIV 14 min (11–21.5) vs. HBP 6 min (4.5–10) vs. LBBP 4.5 min (3.1–7.5); *p* < 0.001).

In most BIV patients LV lead was positioned in the lateral (5 patients) or posterolateral vein (4 patients), only 3 patients had the lead implanted in the antero-lateral vein. Nine patients had undergone AVNA during the same hospitalization, while the remaining 3 AVNAs were performed within 21 days after BIV pacemaker implantation. One procedure-related adverse event was documented in BIV group. In one patient slight quadripolar LV lead dislodgement occurred during AVNA, however, BIV pacing with previous ECG morphology was successfully restored after device reprograming.

Selective HBP was achieved in 11 (40.7%) patients. All patients received back-up RV pacing lead. AVNAs were performed immediately after device implantation. Acute increase of HBP lead threshold after AVNA was registered in 1 patient (from 1.8 to 3.2 V at 1 ms). Nevertheless, the capture of the conduction system was not compromised and the lead revision was not necessary. Two HBP implantations were performed in patients with cardiogenic shock and temporal circulatory support with veno-arterial extracorporeal membrane oxygenation (ECMO) or intra-aortic balloon pump (IABP). 

Selective LBBP was achieved in all 10 patients. There were no complications during device implantation or AVNA.

### 3.3. Electrical Parameters

**Figure 2:**
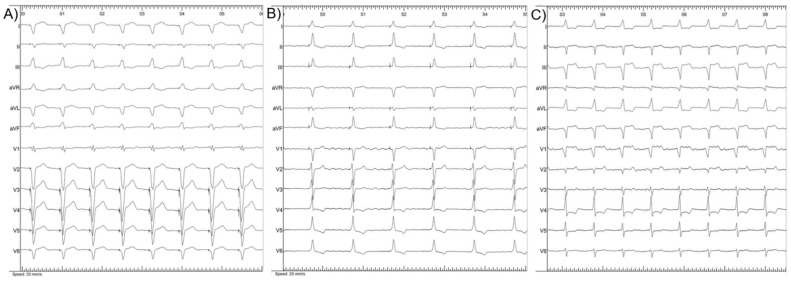
Post-procedural 12-lead body surface ECG of biventricular pacing (**A**), His bundle pacing (**B**) and left bundle branch pacing (**C**).

Before the procedure, there were no significant differences in QRS duration according to pacing modality (*p* = 0.145). Post-procedural QRS duration was significantly shorter (*p* < 0.001) in CSP than in BiV pacing group (Table 2). Three post-procedural electrocardiograms (ECGs) are presented in Figure 2.

Significantly lower pacing thresholds were achieved in LBBP than in HBP or BiV groups (*p* = 0.006) (Table 2). Due to the increase in capture tresholds (from 2.5 to 4 V and from 2 to 6 V at 1 ms) requiring reprogramming to back-up RV lead pacing, permanent HBP was achieved in 25 patients (92.6%). Only patients with successful permanent HBP were included in the analysis of clinical and echocardiographic outcomes. In the patient implanted on IABP support acute HBP threshold 4 V at 1 ms which decreased to 3.25 V at 1 ms was considered acceptable. Battery replacement with de-novo LBBP lead implantation was performed after 3 years. In the remaining patients no back-up RV pacing was required and pacing thresholds remained stable during follow-up (1.25 V (0.875–1.9) vs. 0.75 V (0.5–1.875), *p* = 0.370). Stable pacing thresholds during follow-up were also noted in BiV (1.4 V (1.1–1.75) vs. 1.5 V (1–1.625), *p* = 0.765) and LBBP groups (0.8 V (0.5–1.1) vs. 0.8 V (0.5–1), *p* = 0.799). Spontaneous conversion to sinus rhythm was detected in the only patient with implanted atrial lead. Consequently, pacemaker was reprogrammed to DDD mode. The electrical parameters recorded from implanted devices are summarized in Table 3.

### 3.4. Clinical Outcomes

Before the procedure, the median NYHA class in all three groups was 3 (*p* = 0.175 for intergroup comparison) (Table 4). When compared to baseline, there was no significant change in NYHA class in BIV group. (*p* = 0.096). While 6 patients (46.2%) in BiV group improved for 1 NYHA class, 2 patients (15.4%) declined for 1 NYHA class. Contrary, significant symptomatic improvement according to NYHA class was achieved in both CSP groups (*p* < 0.001 for HBP, *p* = 0.008 for LBBP). Eighteen patients (72%) in the HBP and 8 patients (80%) in the LBBP group improved for at least 1 functional class and no deterioration was detected. 

Heart failure therapy did not change during follow-up, however in HBP group we observed a significant decrease in the use of loop diuretics (*p* = 0.014), as they were discontinued in 6 (40%) out of 15 patients receiving them at baseline. Loop diuretics were discontinued in 2 (28.6%) out of 7 patients in both BIV and LBBP groups, not reaching statistical significance. Digoxin and amiodarone were discontinued in all patients. 

The median baseline N-terminal prohormone of brain natriuretic peptid (NT-proBNP) was 2417 pg/mL (1423–3635) and did not differ between groups (*p* = 0.339). At follow-up, there was a significant reduction of NT-proBNP in both CSP groups (HBP: from 2800 pg/mL (1257–5977) to 1472 pg/mL (904–2113), *p* = 0.001; LBBP: from 2689 pg/mL (1603–5710) to 1632 pg/mL (861–5028), *p* = 0.047). In contrast, no significant decrease was observed in the BiV group (1908 pg/mL (1215–2825) vs. 1856 pg/mL (1195–2505), *p* = 0.331). Similarly, at 6 months there was an improvement in estimated glomerular filtration rate (eGFR) in the both CSP groups (HBP: *p* = 0.001; LBBP: *p* = 0.033), however no significant improvement of eGFR was noted in the BiV group (*p* = 0.349) (Table 4). 

Three patients (2 in BiV group and 1 in HBP group) died during follow-up. While one death in the BIV group was related to progressive heart failre, other 2 deaths were non-cardiac.

### 3.5. Echocardiographic Outcomes

**Figure 3:**
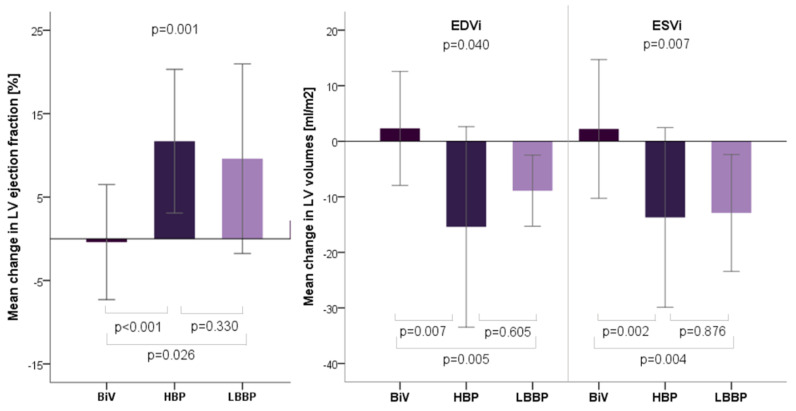
Comparison of mean (±SD) changes in echocardiographic left ventricular volumes and ejection fraction between baseline and follow-up values for all pacing modalities. Upper *p* value (ANOVA) determines whether differences between the means of all 3 groups are statistically significant. *p* values comparing each two groups (*t*-test) are added at the bottom. Legend: BIV: biventricular pacing; HBP: His bundle pacing; LBBP: left bundle branch pacing; LV: left ventricle; LVEDVi: left ventricular end-diastolic volume indexed to body surface area; LVESVi: left ventricular end-systolic volume indexed to body surface area.

While there were no significant differences in LVEF (*p* = 0.149) and indexed LV end diastolic volume (LVEDVi) (*p* = 0.06) at the baseline, initial indexed LV systolic volume (LVESVi) was significantly smaller in HBP group (*p* = 0.033). During follow-up LVEF improved in both HBP (from 39% (31–46) to 49% (42–58); *p* < 0.001) and LBBP groups (from 28% (20–43) to 40% (31–44); *p* = 0.041), but remained unchanged in the BIV group (38% (35–40) vs. 37% (35–41), *p* = 0.916). As for LVEF, we observed consistent changes in LV volumes. Both indexed LV volumes decreased in HBP and in LBBP groups. In contrast, there was no significant change in indexed LV volumes in BiV group (Table 5). Comparison of mean changes in echocardiographic parameters are presented in Figure 3.

## 4. Discussion

The present study assessed different permanent pacing modalities in a subgroup of patients with HF and narrow QRS who underwent AVNA. There were two major findings. First, AVNA combined with CSP was feasible and safe in high percentage of patients with rate refractory AF and HF. Second, CSP was associated with superior clinical and echocardiographic improvement compared to BiV pacing. 

### 4.1. Procedures Assessment

BiV pacing is an established treatment option for patients undergoing the “pace and ablate” strategy [6,7,8]. Recently reported high acute success rates of HBP and LBBP made these two modalities a potential alternative to BiV [24]. In the line with these reports, the present study demonstrated successful permanent CSP pacemaker implantation combined with AVNA in all enrolled patients. However, there are several hesitations that limit wider clinical application of CSP in routine clinical practice. Especially HBP is related to technical challenges with prolonged learning curve, longer fluoroscopy duration, higher and unstable capture thresholds with early battery depletion [11,25]. Recently published meta-analysis have demonstrated that LBBP might overcome these deficiencies [25]. In our study, LBBP appeared to result in shorter fluoroscopy time duration, less complications during AVNA and lower pacing thresholds compared to HBP group and BiV group. Namely, LBBP lead is screwed deep within the LV septum and close to the myocardial tissue, thus stimulating not only the specialized conduction system but also the deep myocardium of the interventricular septum [24]. In accordance with previous published literature, pacing thresholds in our study remained stable during short-term follow-up in both CSP groups [24,25]. Nonetheless, rise in capture threshold was registered in 2 patients with HBP, which required programming to asynchronous RV pacing, therefore favoring LBBP over HBP.

### 4.2. Electocardiographic and Echocardiographic Outcomes

Previously published studies have failed to show substantial benefit of BiV in HF patients with narrow QRS (≤130 ms) [26]. In the present study, BiV pacing resulted in significantly wider post-procedural QRS complexes compared to CSP. These results indicate that BiV pacing still causes abnormal cardiac activation with consequent worsening of electrical dyssynchrony and LV function. 

The echocardiographic outcomes of BiV pacing have been inconsistently reported in the published literature. The Ablate and Pace in Atrial Fibrillation (APAF) trial showed significant improvement of LV function in patients with symptomatic AF who undergone BiV pacemaker implantation combined with AVNA [7]. In addition, later published meta-analyses showed LVEF improvement of only 2% and LV volume decrease by merely 2.65 mL. However, among the studies that reported baseline QRS duration, only 50% of patients had QRS durations of more than 120 ms [5]. Similar conclusion could be made from Ablate and Pace in Atrial Fibrillation plus Cardiac Resynchronisation Therapy (APAF-CRT) trial, where no clear advantages of BiV pacing combined AVNA over pharmacological treatment were observed in patients with narrow QRS and mid-range LVEF [8]. Consequently, we can assume that BiV pacing would be more efficient in patients with wide QRS. In the line with the previous statement are the results of Khan et al. study, which compared pulmonary vein isolation and BiV pacing combined with AVNA in patients with narrow QRS. A decrease of 1 ± 4% in LVEF was noticed in the BiV group [27]. Only patients with narrow QRS were included in our study and echocardiographic outcomes of BiV pacing resemble those mentioned in study by Khan et al. Slight, although not statistically significant deterioration of LVEF was observed (0.5 ± 6.7%), with no significant change in indexed LV volumes. In contrast, LVEF improved significantly in HBP group (mean change 14 ± 12.3%) and LBBP group (mean change 9.6 ± 11.4). Furthermore, both CSP modalities resulted in positive left ventricular remodelling with end diastolic volume and end systolic volume reduction. Although, there was no significant difference between improvement in both CSP modalities, slightly inferior outcomes in LBBP group could be attributed to shorter median follow-up in this group compared to HBP. The results in present study are in accordance with previous published literature. Su et al. [28] in prospective observational study reported LVEF improvement from 44.9  ±  14.9% to 57.6  ±  12.5% and Huang et al. [12] reported LVEF improvement from 32.2 ± 4.8 to 57.2 ± 8.7% in patients with reduced baseline LVEF after HBP and AVNA. To the best of our knowledge, the only study evaluating echocardiographic outcomes after LBBP combined with AVNA is by Wang et al. [17], although they reported combined results of HBP and LBBP. Significant decrease in LV endsystolic volume and an increase in LVEF were documented [17]. 

### 4.3. Clinical Outcomes

Significant symptomatic improvement was achieved in CSP groups: 72% patients in HBP and 80% in LBBP group improved at least 1 NYHA class. The number of patients taking loop diuretics at follow-up decreased significantly only in HBP group. As mentioned above, this difference could be explained with longer median follow-up time compared to LBBP group. In the study by Wang et al. [17], which compared pharmacological therapy and “pace and ablate” strategy in patients with permanent AF and ICD, CSP resulted in less HF hospitalizations, reduced use of diuretics and improved NYHA classification. Huang et al. [12] noted that NYHA classification improved to 1.4 ± 0.4 after 1 year of HBP from the baseline 2.9 ± 0.6 in patients with reduced LVEF. Symptomatic benefits of CSP modalities combined with AVNA were also demonstrated in similar studies [19,29]. The present study did not demonstrate significant NYHA class or diuretics intake decline in BiV group, short follow-up did not allow deeper evaluation of potential clinical benefits. When comparing BiV and RV pacing, BiV pacing did not improve 6 min walk test or quality life score. However, it did lead to non-significant reduction in mortality and a significant reduction in hospitalizations for heart failure compared with RV pacing [5]. Compared with drug therapy, similar observations were found in the APAF-CRT study. Patients with severely reduced LVEF exhibited significant symptomatic benefit after AVNA and BiV pacing, yet no clear benefit regarding mortality [8]. All three pacing modalities provide regularization of heart rhythm and definite rate control, therefore, physiological cardiac activation, superior LV function improvement and positive remodelling could explain greater alleviation of symptoms in CSP group compared to BiV group after medium-term follow-up. Therefore, it is reasonable to assume that CSP (HBP or LBBP) in conjunction with AVNA could be a promising alternative in refractory AF patients with reduced EF and narrow QRS. However, prospective randomised studies with longer follow-up are warranted to clarify the optimal pacing modality in combination with AVNA. 

### 4.4. Study Limitations

Non-randomized retrospective single-center study design with low number of enrolled patients limits the strength of our findings. Furthermore, shorter follow-up in LBBP group might have affected the echocardiographic and clinical outcomes compared to HBP. In addition, mid-term follow-up may underestimate the concern of unpredictable increase in HBP or LBBP thresholds, loss of capture or lead interventions. Long-term benefits and safety of permanent CSP warrant further evaluation. Relatively long post-implant QRS in BiV group might have led to inferior echocardiographic improvement compared to the CSP modalities. Additional pre-procedural imaging, intra-operative measurements (ventricular activation times, scar identification, etc.), and device optimisation could have yielded shorter BiV paced QRS intervals. However, in patients with narrow QRS undergoing BiV pacing and AVNA, LV lead positioning principally relies on the anatomical approach due to short interventricular delays, thus limiting this pacing method even further in case of unfavorable venous anatomy. As only patients with reduced LVEF (EF < 50%) and narrow QRS (<120 ms) were included, the results cannot be extrapolated to other subgroups of patients undergoing AVNA. Additional limitation is the relatively small number of included patients that contributed to some differences in baseline characteristics, which were however not statistically significant. Findings should be interpreted with caution and need to be confirmed with adequately large-scale randomized clinical trials of diverse groups of HF patients with long-term follow-up.

## 5. Conclusions

In symptomatic AF patients with reduced LVEF and narrow QRS, CSP modalities showed superior symptomatic and echocardiographic improvement compared to BiV pacing after AVNA. While LBBP offered lower and more stable pacing parameters, there were no differences in clinical outcomes and echocardiographic remodelling when compared to HBP. Therefore, these findings could support wider clinical adoption of CSP in conjunction with AVNA. However, the technique’s widespread adaptation needs further validation to ascertain its safety and efficacy in randomized clinical trials with longer follow-up. 

## Figures and Tables

**Table 1:** Baseline characteristics of patients by pacing modality.

	BiV (*n* = 13)	HBP (*n* = 27)	LBBP (*n* = 10)	*p* Value
Characteristics				
Age [years]	70 (67–73.5)	71 (62–75)	69 (67–78)	0.888
Male sex	7 (53.8%)	10 (37.0%)	7 (70.0%)	0.196
QRS [ms]	98 (±7)	100 (±13)	105 (±15)	0.145
Heart rate [bpm]	128 (113–137)	133 (123–141)	127 (97–132)	0.278
Atrial flutter	2 (15.4%)	6 (22.2%)	3 (30.0%)	0.815
LVEF [%]	38 (35–40)	39 (30–45)	28 (20–42)	0.135
LAVI [mL/m2]	55 (±11)	55 (±11)	59 (±14)	0.975
Initial median NYHA class	3	3	3	0.175
Comorbidities				
AH	9 (69.2%)	17 (63.0%)	8 (80.0%)	0.665
Diabetes	3 (23.1%)	8 (29.6%)	2 (20.0%)	0.914
CAD	6 (46.2%)	6 (22.2%)	4 (40.0%)	0.260
Medication				
ACEi/ARB/ARNI	9 (69.2%)	20 (74.1%)	6 (60.0%)	0.716
MRA	7 (53.8%)	9 (33.3%)	5 (50.0%)	0.434
BB	13 (100%)	25 (92.6%)	9 (90.0%)	0.767
Digoxin	5 (38.5%)	7 (25.9%)	2 (20.0%)	0.640
Amiodarone	2 (15.4%)	6 (22.2%)	1 (10.0%)	0.887
Anticoagulation	13 (100%)	25 (92.6%)	10 (100%)	1
Loop diuretic	7 (53.8%)	17 (63.0%)	7 (70.0%)	0.794

BIV: biventricular pacing; HBP: His bundle pacing; LBBP: left bundle branch pacing; LVEF: left ventricle ejection fraction; LAVI: left atrial volume index; AH: arterial hypertension; CAD: coronary artery disease; ACEi: angiotensin-converting enzyme inhibitor; ARB: angiotensin II receptor blocker; ARNI: angiotensin receptor neprilysin inhibitor; MRA: mineralocorticoid receptor antagonist; BB: beta blocker.

**Table 2:** Electrocardiographic characteristics at baseline and follow-up and initial device parameters by pacing modalities.

	BiV (*n* = 13)	HBP (*n* = 27)	LBBP (*n* = 10)	*p* Value
Electrocardiographic characteristics				
Baseline QRS [ms]	98 (±7)	100 (±13)	98 (±7)	0.145
Post-implant QRS [ms]	172 (±13)	105 (±17))	127 (±13)	<0.001
Lead measurements				
Initial CSP/LV threshold [V]	1.4 (1.1–1.75)	1.25 (1–2)	0.8 (0.5–1.1)	0.006
Initial CSP/LV impedance [ohm]	760 (±229)	526 (±87)	750 (±77)	<0.001

Thresholds in BiV and HBP groups were measured at 1ms, in LBBP group at 0.5 ms. BiV: biventricular pacing; HBP: His bundle pacing; LBBP: Left bundle branch pacing; CSP: conduction system pacing; LV: left ventricle.

**Table 3:** Pacing parameters by pacing modality at baseline and follow-up.

	BiV (*n* = 13)	HBP (*n* = 25) *	LBBP (*n* = 10)
Initial CSP/LV threshold [V]	1.4 (1.1–1.75)	1.25 (0.875–1.9)	0.8 (0.5–1.1)
Follow-up CSP/LV threshold [V]	1.5 (1–1.625)	0.75 (0.5–1.875)	0.8 (0.5–1)
*p* value: initial vs. follow-up	0.765	0.370	0.799
Initial CSP/LV impedance [ohm]	760 (±229)	526 (±90)	749 (±77)
Follow-up CSP/LV impedance [ohm]	682 (±161)	465 (±72)	594 (±137)
*p* value: initial vs. follow-up	0.142	0.008	0.002

BiV: biventricular pacing; HBP: His bundle pacing; LBBP: Left bundle branch pacing; CSP: conduction system pacing; LV: left ventricle. * 2 patients were switched to RV septal pacing during follow-up. Thresholds in BiV and HBP groups were measured at 1ms, in LBBP group at 0.5 ms.

**Table 4:** Clinical and laboratory features of patients by pacing modality at baseline and follow-up.

	BiV (*n* = 13)	HBP (*n* = 25)	LBBP (*n* = 10)	*p* Value—Comparing Groups
NYHA class				
Initial median NYHA class	3	3	3	0.175
Nb. in NYHA class 2	1 (7.7%)	2 (8.0%)	4 (40.0%)	
Nb. in NYHA class 3	11 (84.6%)	18 (72.0%)	5 (50.0%)	
Nb. in NYHA class 4	1 (7.7%)	5 (20.0%)	1 (10.0%)	
Follow-up median NYHA class	3	2	2	0.059
Nb. in NYHA class 1	0	5 (20.0%)	4 (40.0%)	
Nb. in NYHA class 2	6 (46.2%)	15 (60,0%)	5 (50.0%)	
Nb. in NYHA class 3	6 (46.2%)	5 (20.0%)	1 (10.0%)	
Nb. in NYHA class 4	1 (7.7%)	0	0	
*p* value: initial vs. follow-up	0.096	<0.001	0.008	
Loop diuretics				
Initial	7 (53.8%)	17 (63.0%)	7 (70.0%)	0.8
Follow-up	6 (46.2%)	9 (33.3%)	6 (60.0%)	0.403
*p* value: initial vs. follow-up	0.564	0.014	0.564	
NT-proBNP [pg/mL]				
Initial	1908 (1215–2825)	2800 (1257–5977)	2689 (1603–5710)	0.339
Follow-up	1856 (1195–2505)	1472 (904–2113)	1632 (861–5028)	0.599
*p* value: initial vs. follow-up	0.311	0.001	0.047	
eGFR [mL/min/1.73 m^2^]				
Initial	58 (51–62)	52 (45–61)	66 (35–84)	0.240
Follow-up	60 (49–66)	67 (55–73)	79 (41–90)	0.214
*p* value: initial vs. follow-up	0.349	0.001	0.033	

BiV: biventricular pacing; HBP: His bundle pacing; LBBP: Left bundle branch pacing; NYHA: New York Heart Association; NT-proBNP: N-terminal prohormone of brain natriuretic peptide; GFR:glomerular filtration rate.

**Table 5:** Echocardiographic outcomes of patients by pacing modality at baseline and follow-up.

	BiV (*n* = 13)	HBP (*n* = 25) *	LBBP (*n* = 10)
Initial LVEF [%]	38 (35–40)	39 (31–46)	28 (20–43)
Follow-up LVEF [%]	37 (35–41)	49 (42–58)	40 (31–44)
*p* value: initial vs. follow-up	0.916	<0.001	0.041
Initial LVEDVi [mL/m^2^]	82 (±17)	72 (±21)	89 (±22)
Follow-up LVEDVi [mL/m^2^]	84 (±19)	61 (±18)	81 (±21)
*p* value: initial vs. follow-up	0.509	0.006	0.002
Initial LVESVi [mL/m^2^]	51 (±12)	45 (±18)	63 (±21)
Follow-up LVESVi [mL/m^2^]	53 (±14)	32 (±13)	50 (±18)
*p* value: initial vs. follow-up	0.551	<0.001	0.004

Legend: BiV: biventricular pacing; HBP: His bundle pacing; LBBP: Left bundle branch pacing; LVEF: left ventricular ejection fraction; LVEDVi: left ventricular end-diastolic volume indexed to body surface area; LVESVi: left ventricular end-systolic volume indexed to body surface area. * 2 patients were switched to RV septal pacing during follow-up.

## Data Availability

The data presented in this study are available upon request from the corresponding author and are not publicly available due to ethical issues.

## Abbreviations

CSP	conduction system pacing
HBP	His-bundle pacing
LBBP	left bundle branch pacing
BiV	biventricular
RV	right ventricle
LV	left ventricle
AVNA	atrioventricular node ablation
AF	atrial fibrillation
HF	heart failure
ESC	European Society of Cardiology
LVEF	left ventricle ejection fraction
LAVI	left atrial volume index
LVEDVi	LV end dyastolic volume index
LVESVi	LV end systolic volume index
NYHA	New York Heart Association
ICD	implantable cardioverter-defibrillator
ECG	electrocardiogram
NT-proBNP	N-terminal prohormone of brain natriuretic peptid (NT-proBNP)
GFR	glomerular filtration rate
